# Microprotein, macro-effect: DWORF as a therapeutic strategy in heart failure

**DOI:** 10.3389/fcell.2026.1864847

**Published:** 2026-07-01

**Authors:** Joseph P. Verry, Catherine A. Makarewich

**Affiliations:** 1 The Heart Institute, Division of Molecular Cardiovascular Biology, Cincinnati Children’s Hospital Medical Center, Cincinnati, OH, United States; 2 Medical Scientist Training Program, University of Cincinnati College of Medicine, Cincinnati, OH, United States; 3 Pathobiology and Molecular Medicine Graduate Program, University of Cincinnati College of Medicine, Cincinnati, OH, United States; 4 Department of Pediatrics, University of Cincinnati College of Medicine, Cincinnati, OH, United States

**Keywords:** calcium handling, DWORF, gene therapy, heart failure, microprotein, sarcoplasmic reticulum, SERCA2a, STRIT1

## Abstract

Heart failure (HF) is a global health crisis and remains a leading cause of morbidity, mortality, and healthcare expenditure despite substantial advances in pharmacological and device-based therapy. Impaired sarcoplasmic reticulum (SR) Ca^2+^ cycling, driven largely by reduced activity of the SR Ca^2+^-ATPase 2a (SERCA2a), is a hallmark molecular feature of the failing myocardium. The SERCA2a-phospholamban (PLN) regulatory axis has long attracted therapeutic interest, however, clinical translation of SERCA2a gene therapy has been inconsistent, and strategies targeting PLN raise safety concerns, as complete PLN loss is lethal in humans. Dwarf Open Reading Frame (DWORF; gene name *STRIT1*) is a 34–35 amino acid single-pass transmembrane microprotein and the only known endogenous positive regulator of SERCA2a within the SERCA-regulin family. In contrast to inhibitory regulins including PLN, sarcolipin (SLN), myoregulin (MLN), endoregulin (ELN), and another-regulin (ALN), DWORF enhances SERCA2a activity by increasing its catalytic turnover and displacing PLN from a shared regulatory binding site, thereby promoting Ca^2+^ reuptake into the SR. Structurally, a proline at position 15 (Pro15) introduces a critical kink that separates DWORF’s N-terminal amphipathic helix from its C-terminal transmembrane domain, with disruption of this feature abolishing DWORF’s activating properties and converting DWORF into a PLN-like inhibitor. DWORF expression is restricted to ventricular myocardium and slow-twitch skeletal muscle, increases postnatally with cardiac maturation, and is consistently reduced in heart failure models. Across multiple preclinical models including dilated cardiomyopathy, Duchenne muscular dystrophy cardiomyopathy, pressure overload-induced heart failure, PLN-R14del cardiomyopathy, and ischemia-reperfusion injury, DWORF gene therapy improves Ca^2+^ handling, reduces fibrosis, and restores mitochondrial energetics, even when administered after established disease. The compact coding sequence of DWORF is ideally suited for adeno-associated virus delivery, enabling efficient packaging and potential multiplexing strategies. Together, these features position DWORF as a mechanistically distinct and physiologically robust strategy for enhancing SERCA2a function. This review summarizes the molecular mechanisms underlying DWORF activity, evaluates preclinical therapeutic evidence, and discusses key translational considerations, including species differences, safety, and remaining knowledge gaps that must be addressed to advance DWORF-based therapy toward clinical application in heart failure.

## 1 Introduction

Heart failure (HF) affects more than 64 million people worldwide and is associated with high rates of hospitalization, reduced quality of life, and 5-year mortality exceeding 50% even with modern medical therapy (Bozkurt et al., 2025; GBD, 2017 Disease and Injury Incidence and Prevalence Collaborators, 2018; Osenenko et al., 2022). Despite meaningful advances in neurohormonal blockade, device therapy, and emerging pharmacological agents, the fundamental defect in cardiomyocyte Ca^2+^ handling that drives contractile dysfunction and energetic failure in the failing heart remains incompletely addressed by current standard-of-care treatment (Gorski et al., 2015; Siri-Angkul et al., 2021).

Excitation-contraction coupling in cardiomyocytes relies on tightly controlled Ca^2+^ flux between the extracellular space, cytosol, and sarcoplasmic reticulum (SR) (Gilbert et al., 2020). Membrane depolarization during the cardiac action potential opens L-type Ca^2+^ channels, allowing a small influx of Ca^2+^ that triggers a much larger release of Ca^2+^ from ryanodine receptors (RyR2) on the SR through the process of Ca^2+^-induced Ca^2+^ release (Bers, 2006; Marx et al., 2000). The resulting rise in cytosolic Ca^2+^ binds to troponin C on the thin filament, initiating conformational changes in the troponin-tropomyosin complex that permit actin-myosin cross bridge formation and drive contraction (Ko et al., 2022). Relaxation is achieved primarily through reuptake of Ca^2+^ into the SR by the ATP-dependent pump SERCA2a, with a smaller contribution from Ca^2+^ extrusion across the sarcolemma via the Na^+^/Ca^2+^ exchanger (NCX) (Bers, 2002).

SERCA2a activity is tightly regulated by phospholamban (PLN), a small transmembrane microprotein that inhibits SERCA2a in its dephosphorylated state by lowering the pump’s apparent affinity for Ca^2+^ (MacLennan and Kranias, 2003). During β-adrenergic stimulation, activation of protein kinase A (PKA) and Ca^2+^/calmodulin-dependent protein kinase II (CaMKII) leads to phosphorylation of PLN at Ser16 and Thr17, respectively (Glaves et al., 2011). Phosphorylation relieves PLN-mediated inhibition of SERCA2a and accelerates SR Ca^2+^ reuptake, thereby enhancing relaxation and increasing the rate at which cardiomyocytes cycle Ca^2+^ during periods of increased workload (Brittsan et al., 2000). When not bound to SERCA2a, PLN is sequestered by the formation of homopentamers in the SR membrane, which is stabilized by phosphorylation (Cornea et al., 1997; Kimura et al., 1997). This dynamic regulatory mechanism enables the heart to rapidly adjust contractility in response to changing physiological demand (Funk et al., 2023).

In heart failure, multiple components of the Ca^2+^-handling machinery become maladaptively remodeled (Hasenfuss et al., 1994). SERCA2a expression and activity are typically reduced, while NCX expression is often increased, shifting Ca^2+^ removal away from SR reuptake toward sarcolemmal extrusion (Gorski et al., 2015; Hasenfuss et al., 1994; Pott et al., 2011). As a consequence, SR Ca^2+^ content declines, systolic Ca^2+^ transients are diminished, relaxation becomes slower, and contractile reserve is impaired (Bers, 2006; Terracciano et al., 2004). Chronic neurohormonal signaling further disrupts Ca^2+^ cycling by altering PLN expression, phosphorylation status, and its interaction with SERCA2a, thereby maintaining an inhibitory tone on the pump (Luo and Anderson, 2013). Together, these alterations contribute to both systolic dysfunction and diastolic abnormalities that characterize failing myocardium.

Because of its central role in Ca^2+^ cycling, the SERCA-PLN regulatory axis has long been considered an attractive therapeutic target (Zhihao et al., 2020). Prior strategies have included SERCA2a overexpression via gene therapy and approaches aimed at reducing PLN-mediated inhibition, either through genetic ablation or by enhancing PLN phosphorylation (Njegic et al., 2020). Although these interventions have shown encouraging effects in preclinical models, clinical translation has been challenging. Limitations include vector design constraints, incomplete functional rescue, and safety considerations such as arrhythmia or unintended effects in non-cardiac tissues (Yla-Herttuala, 2015). In this context, cardiac microproteins that positively modulate SERCA function, such as Dwarf Open Reading Frame (DWORF), represent a mechanistically distinct strategy to restore Ca^2+^ cycling while potentially preserving physiological control of pump function (Elbatreek and Lefer, 2025). Here, we review the molecular biology of DWORF, evaluate preclinical therapeutic evidence across multiple disease models, and identify critical gaps that must be addressed to advance DWORF-based gene therapy toward clinical application.

## 2 The SERCA-Regulin family and DWORF

### 2.1 Cardiac microproteins and the SERCA-regulin family

A growing family of conserved, small transmembrane microproteins has emerged as key regulators of SERCA activity (Anderson et al., 2016; Magny et al., 2013). These proteins, collectively referred to as the “SERCA-regulins” include phospholamban (PLN), sarcolipin (SLN), myoregulin (MLN), endoregulin (ELN), and another-regulin (ALN). Each contains a single transmembrane helix but differs in tissue distribution, regulatory properties, and SERCA isoform preference (Coughlin and Makarewich, 2025; Rathod et al., 2021). Functionally, the SERCA-regulins inhibit SERCA activity by reducing the pump’s apparent affinity for Ca^2+^ and/or alter its catalytic turnover rate. Through these mechanisms, the SERCA-regulins act as tunable rheostats on Ca^2+^ reuptake that help tailor Ca^2+^ cycling dynamics to the physiological requirements of different cell types. For example, PLN predominantly regulates SERCA2a in cardiac muscle, while SLN and MLN play more prominent roles in skeletal muscle (Anderson et al., 2015; Chambers et al., 2022). This regulatory network allows fine control of Ca^2+^ handling, balancing contractile performance with cellular energetic demands. Within this family, DWORF is unique in that it functions as a positive regulator of SERCA2a (Nelson et al., 2016). Key features of the SERCA-regulin family, including DWORF, are summarized in Table 1.

**TABLE 1:** Structural and functional comparison of the SERCA-regulin family.

Feature	PLN	SLN	MLN	ELN	ALN	DWORF
Length (aa; human)	52	31	46	62	66	35
Primary Tissue Expression	Cardiac muscle (atria and ventricles)	Skeletal muscle; atria	Skeletal muscle	Endothelial and epithelial tissues	Ubiquitous (all tissues)	Ventricular myocardium; slow-twitch skeletal muscle
Major SERCA Isoform(s)	SERCA2a	SERCA1a, SERCA2a	SERCA1a	SERCA3	SERCA2b	SERCA2a
Functional Effect on SERCA	Inhibitory; reduces apparent Ca^2+^ affinity	Inhibitory; reduces apparent Ca^2+^ affinity and Vmax	Inhibitory; reduces Vmax	Inhibitory; reduces Vmax	Inhibitory; reduces apparent Ca^2+^ affinity and Vmax	Activating; increases SERCA Vmax and displaces inhibitory regulins
Key Structural/Sequence Features	Cytoplasmic regulatory domain; transmembrane inhibitory helix; Ser16/Thr17 phosphorylation sites (PKA/CaMKII)	Single transmembrane helix; Thr5 phosphorylation site (CaMKII)	Conserved trans-membrane regulin motif; structural similarity to PLN/SLN	Extended luminal/cytosolic domains; single-pass transmembrane regulin structure	Conserved regulin transmembrane motif; Ser19 phosphorylation site (PKC)	Pro15-induced kink separating amphipathic and transmembrane domains; GxxxG oligomerization motif
Oligomerization	Stable pentamers and monomers	Monomeric and oligomeric forms reported	Oligomerization less well defined	Unknown/poorly characterized	Unknown/poorly characterized	Monomers, dimers, and tetramers reported
Physiological/Disease Relevance	Central regulator of cardiac contractility; mutations linked to dilated and arrhythmogenic cardiomyopathy	Regulates skeletal muscle and atrial Ca^2+^ handling	Regulates skeletal muscle performance and Ca^2+^ cycling	May regulate Ca^2+^ handling in non-muscle secretory tissues	Implicated in adipocyte and cardiac Ca^2+^ regulation; expression altered in metabolic stress states	Downregulated in heart failure; therapeutic benefit demonstrated in multiple preclinical HF models
References	Minamisawa et al. (1999), MacLennan and Kranias (2003), Haghighi et al. (2003), Vangheluwe et al. (2005), Periasamy et al. (2008), Glaves et al. (2011), Anderson et al. (2016), Rathod et al. (2021)	Vangheluwe et al. (2005), Periasamy et al. (2008), Bhupathy et al. (2009), Anderson et al. (2016), Rathod et al. (2021)	Anderson et al. (2015), Anderson et al. (2016), Rathod et al. (2021)	Anderson et al. (2016), Rathod et al. (2021)	Anderson et al. (2016), Rathod et al. (2021), Hassel et al. (2024), Auger et al. (2025)	Nelson et al. (2016), Makarewich et al. (2018), Singh et al. (2019), Makarewich et al. (2020), Li et al. (2021), Rathod et al. (2021), Reddy et al. (2022), Fisher et al. (2025)

Summary of conserved SERCA-regulating microproteins, including amino acid (aa) length (human isoforms), primary tissue distribution with corresponding SERCA isoforms, functional effects on SERCA activity, key structural features, oligomerization properties, and known physiological or disease relevance. Although all SERCA-regulins share a conserved single-pass transmembrane architecture, they exhibit substantial diversity in tissue specificity, regulatory mechanisms, and physiological function. DWORF is unique among the family as the only known endogenous positive regulator of SERCA2a.

### 2.2 DWORF: discovery and gene structure

Unlike the other regulins that inhibit pump activity, DWORF enhances SERCA2a function and promotes Ca^2+^ reuptake into the SR. The DWORF locus was originally annotated as a noncoding region until subsequent open reading frame analysis revealed a conserved coding sequence encoding a small peptide (Nelson et al., 2016). The mature protein is 34–35 amino acids in length, with the 35-amino acid isoform produced by alternative splicing at the exon two boundary, resulting in the inclusion of a single alanine residue at the splice junction. The DWORF protein has an apparent molecular weight of ∼3.8–4 kDa. This combination of small size, strong evolutionary conservation, and functional activity opposite that of the inhibitory regulins positions DWORF as a conceptually distinct component of the SERCA2a regulatory network (Coughlin and Makarewich, 2025; Nelson et al., 2016).

### 2.3 Localization and topology

**FIGURE 1:**
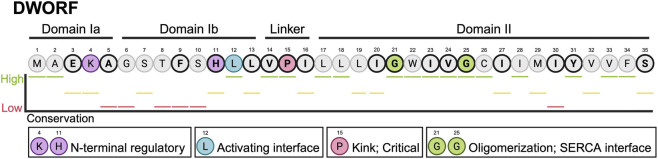
Schematic of human DWORF sequence including Domain Ia (M1-A5), Domain Ib (G6-L13), Linker domain (V14-I16), and Domain II (L17-S35). Residues in bold are sites of known human variants identified through genome or exome sequencing. Ranking of conservation is annotated as low (red), medium (yellow), or high (green). Important residues are highlighted including: K4 and H11 which are important for N-terminal regulatory function, L12 which is important for the SERCA2a activating interface and aligns with L31 in PLN and L8 in SLN, P15 which is important for DWORF’s proline-induced kink and shown to be critical for overall DWORF function, and G21 and G25 which are part of a GxxxG motif that is important for oligomerization and the SERCA2a interface.

DWORF localizes specifically to the SR membrane in cardiomyocytes, where it co-resides with SERCA2a and other regulins in both the junctional and network SR (Nelson et al., 2016). Topologically, DWORF consists of an N-terminal region (Domain Ia; M1-A5) followed by an amphipathic juxtamembrane helix (Domain Ib; G6-L13) that lies along the cytosolic leaflet of the SR membrane, a linker region centered by a proline at position 15 (V14-I16) and a C-terminal transmembrane helix (Domain II; L17-S35) that spans the membrane (Figure 1) (Fisher et al., 2025; Reddy et al., 2022). Structural and biophysical studies support a helix-linker-helix architecture consisting of an N-terminal amphipathic helix, a short linker segment, and a C-terminal transmembrane helix that engages SERCA2a.

A defining feature of this topology is a proline-induced kink centered at proline 15 (Pro15, P15) within the linker region (Li et al., 2021). This kink separates the two helical segments and helps orient the transmembrane helix relative to the membrane plane and the SERCA binding groove (Reddy et al., 2022). Mutations that reduce or eliminate the kink diminish DWORF’s ability to activate SERCA, indicating that the precise geometry imposed by Pro15 is critical for productive binding and pump activation.

### 2.4 Sequence features and alignment across species

DWORF is highly conserved from jawless fish such as lamprey through higher vertebrates including humans, horses, and dogs, consistent with a strong evolutionary constraint on both its sequence and function (Nelson et al., 2016; Valberg et al., 2019). The N-terminal region contains positively charged residues that likely interact with negatively charged lipid headgroups and/or cytosolic domains of SERCA, whereas the transmembrane segment is enriched in hydrophobic residues that form the core of the helix embedded within the SR membrane.

Two structural features are particularly notable. First, the N-terminal amphipathic helix mirrors the overall organizational principle observed in PLN and SLN, providing a similar membrane proximal regulatory element positioned adjacent to the cytosolic surface (Rathod et al., 2021). Second, the C-terminal transmembrane domain contains a GxxxG motif (Gly21-X-X-X-Gly25 [glycine, G, or Gly]), a sequence motif commonly associated with helix-helix packing interactions in membranes (Fisher et al., 2025; Russ and Engelman, 2000). In DWORF, this motif appears to contribute primarily to the formation of a smooth interface for SERCA binding rather than serving as a classical oligomerization interface (Reddy et al., 2022).

Alignment of DWORF with other regulins reveals that several residues occupy positions analogous to key inhibitory residues in PLN and SLN but are repurposed to support SERCA2a activation. Leu12 in DWORF aligns with Leu31 in PLN and Leu8 in SLN, residues known to be important for inhibitory contacts with SERCA (Kimura et al., 1997; Trieber et al., 2005). Mutating Leu12 partially reduces DWORF‐mediated activation of SERCA (Fisher et al., 2025). Pro15 aligns with Asn34 of PLN and Asn11 of SLN, residues that are central determinants of their inhibitory function (Fisher et al., 2025). Substituting Pro15 with helix-favoring residues such as alanine or asparagine straightens the helix and converts DWORF into a PLN-like inhibitor, producing inhibitory effects on SERCA activity and Ca^2+^ affinity (Fisher et al., 2025). These comparisons highlight how subtle differences in side chain chemistry and helix geometry at conserved positions can convert a regulin from an activator into an inhibitor.

### 2.5 Tissue expression and disease regulation

DWORF expression is restricted to striated muscle and is most abundant in ventricular myocardium and slow-twitch skeletal muscle such as soleus and diaphragm (Nelson et al., 2016; Gibson et al., 2025). In contrast, expression is low or absent in atrial tissue and in prenatal hearts, suggesting that DWORF expression emerges with postnatal maturation of Ca^2+^ handling (Nelson et al., 2016; Gibson et al., 2025). Developmental studies in mice and dogs show that DWORF levels increase after birth, coinciding with the transition to adult-type excitation-contraction coupling and greater reliance on SR-dominated Ca^2+^ cycling (Gibson et al., 2025; Morales et al., 2024; Nelson et al., 2016).

In cardiac disease, DWORF expression is consistently reduced across several models of heart failure and in human failing myocardium (Nelson et al., 2016). Decreases in DWORF mRNA and protein have been reported in mouse models of dilated cardiomyopathy and myocardial infarction, as well as in dystrophic hearts, paralleling reductions in SERCA activity (Makarewich et al., 2020; Makarewich et al., 2018; Morales et al., 2023). Similar patterns are observed in models of Duchenne muscular dystrophy, where DWORF levels decline in both cardiac tissue and select skeletal muscles during disease progression (Gibson et al., 2025). Together, these observations suggest that loss of DWORF may contribute to impaired SERCA function and disrupted Ca^2+^ cycling in heart failure and cardiomyopathy, and that restoring DWORF levels could represent a potential therapeutic strategy.

## 3 Molecular mechanism of DWORF

### 3.1 DWORF as a direct SERCA2a activator

Mechanistic studies in reconstituted systems and heterologous cells converge on the conclusion that DWORF acts as a direct activator of SERCA (Bovo et al., 2024; Cunningham et al., 2025; Fisher et al., 2021; Fisher et al., 2025; Reddy et al., 2022). In purified SERCA1a or SERCA2a reconstitution assays, DWORF increases the maximal turnover rate (Vmax) of the pump by approximately 1.5–1.7-fold relative to SERCA alone, while producing little or no consistent effect on the apparent Ca^2+^ affinity. This pattern suggests that DWORF primarily enhances rate limiting steps of the catalytic cycle rather than altering the equilibrium of Ca^2+^ binding (Cleary et al., 2022). As a consequence, Ca^2+^ transport becomes more efficient, allowing greater Ca^2+^ reuptake into the SR during each catalytic cycle.

Earlier studies using cardiac membrane preparations emphasized an apparent reduction in SERCA K_Ca_ (the Ca^2+^ concentration required for half-maximal activation of SERCA ATPase activity) when DWORF was expressed in the presence of endogenous inhibitory regulins. In these contexts, the affinity shift is largely attributable to relief of PLN/SLN/MLN-mediated inhibition rather than a direct effect of DWORF on Ca^2+^ binding. Indeed, in systems expressing SERCA2a and DWORF alone, K_Ca_ is typically unchanged unless inhibitory regulins are present (Singh et al., 2019). More recent studies using defined heterologous systems expressing RyR2, SERCA2a, PLN, and DWORF together demonstrate that DWORF increases SR Ca^2+^ uptake and SR Ca^2+^ load across a wide range of luminal Ca^2+^ concentrations. These results reinforce the view that DWORF functions as a potent enhancer of SERCA function under physiologically relevant conditions.

### 3.2 PLN competition and displacement

A central component of DWORF’s mechanism of action is its competition with PLN and other inhibitory regulins for binding to SERCA (Li et al., 2021; Makarewich et al., 2018; Nelson et al., 2016). FRET-based binding assays and co-immunoprecipitation experiments indicate an approximately 1:1 stoichiometry in which each SERCA molecule is bound either by PLN or by DWORF, but not both simultaneously. Increasing DWORF expression in cells expressing SERCA2a and PLN reduces PLN-SERCA FRET signals while promoting DWORF-SERCA association, consistent with direct competition for the same binding site (Li et al., 2021). Quantitative binding analyses indicate that DWORF displays a lower dissociation constant (and therefore higher apparent affinity) for SERCA2a than PLN in several systems (Rustad et al., 2023). This higher affinity enables DWORF to displace PLN from the SERCA regulatory cleft and relieve its inhibitory effect. Mutational mapping of SERCA transmembrane helices further supports this model by revealing overlapping binding determinants for PLN and DWORF within the pump (Li et al., 2021; Makarewich et al., 2018). In heart failure, where PLN-SERCA interactions may already be altered because of changes in expression or phosphorylation state, the balance between direct activation and PLN displacement may shift. Under these conditions, the intrinsic ability of DWORF to increase SERCA turnover could become particularly important (Bovo et al., 2024).

### 3.3 Conformation- and Ca^2+^-state-dependent binding

Both the inhibitory and activating SERCA-regulins display preferences for specific conformational states of SERCA (Toyoshima and Nomura, 2002). PLN preferentially binds the E1-ATP state that predominates at low cytosolic Ca^2+^ concentrations. In doing so, PLN stabilizes an inhibited conformation and slows the transition to the Ca^2+^-bound E1 state, thereby reducing the apparent Ca^2+^ affinity of the pump (Cleary et al., 2022). When cytosolic Ca^2+^ rises or PLN becomes phosphorylated, PLN-SERCA interactions weaken, relieving this inhibitory constraint and allowing faster Ca^2+^ binding and transport. DWORF, in contrast, exhibits relatively higher affinity for SERCA states populated at elevated cytosolic Ca^2+^ concentrations, including phosphorylated intermediates such as E1P and E2P that are associated with the rate-limiting steps of the catalytic cycle (Cleary et al., 2022; Toyoshima and Nomura, 2002). This state-dependent binding pattern aligns DWORF function with periods of high Ca^2+^ flux, such as during systole or sustained pacing, when rapid and efficient Ca^2+^ reuptake is required. Together, these complementary binding preferences support a model in which PLN imposes inhibitory control under low cytosolic Ca^2+^ conditions, similar to diastole, whereas DWORF preferentially stabilizes high-throughput SERCA states during periods of increased Ca^2+^ cycling, such as during systole.

### 3.4 Structural requirements for activation versus inhibition

The ability of DWORF to activate rather than inhibit SERCA depends critically on its helix-linker-helix architecture and on specific side-chain identities at key positions (Fisher et al., 2025; Reddy et al., 2022). Mutations that “straighten” the Pro15-induced kink such as Pro15Ala, Pro15Asn, or Pro15Leu, convert the helix into a more continuous, PLN-like conformation and abolish SERCA activation (Fisher et al., 2025; Reddy et al., 2022). In functional assays, these mutants reduce SERCA Vmax and can increase apparent Ca^2+^ affinity and cooperativity in ways that resemble the inhibitory regulins. In addition to Pro15, Leu12 contributes to the activating interface, aligning with characterized inhibitory residues in PLN and SLN (Fisher et al., 2025). Substitution of Leu12 partially impairs DWORF‐mediated activation of SERCA, suggesting that this position participates directly in the activating interaction surface. Lastly, the G-x-x-x-G motif within the transmembrane helix (Gly21 and Gly25) further influences function and oligomerization. Introducing bulkier residues at these positions alters the balance between activation and inhibition and can stabilize dimeric or higher-order oligomeric species. In some double-mutant combinations, these substitutions produce inhibitory behavior (Fisher et al., 2025).

Structural studies indicate that DWORF interacts extensively with the SERCA M2 transmembrane helix while making fewer contacts with the M6 transmembrane helix, in contrast to PLN and SLN, which rely heavily on M6 interactions to impose inhibition (Fisher et al., 2021; Rathod et al., 2021). These observations suggest that subtle differences in helix geometry and contact distribution can determine whether a regulin functions as an inhibitor or an activator.

### 3.5 Oligomerization and interaction with other regulins

The oligomeric state of DWORF in membranes and its relevance to SERCA regulation remain areas of active investigation. Some fluorescence‐based and biochemical studies suggest that DWORF binds SERCA primarily as a monomer, with most DWORF molecules existing in a monomeric pool and only a minor fraction forming dimers or higher oligomers (Makarewich et al., 2018; Singh et al., 2019). Other biophysical analyses, including cross-linking and mutational studies centered on the G-x-x-x-G motif, report that DWORF can form stable dimers and tetramers, with the monomer representing a smaller fraction of the total population (Fisher et al., 2025; Singh et al., 2019).

Beyond homotypic interactions, DWORF may also form hetero-oligomeric complexes with PLN and other regulins. Heterodimerization between DWORF and PLN has been proposed as an additional mechanism through which DWORF reduces the effective pool of PLN available to bind SERCA, complementing direct competition for the SERCA binding site (Phillips et al., 2023). Importantly, mutations that alter DWORF oligomerization do not necessarily abolish SERCA activation, suggesting that monomeric binding to SERCA is sufficient for functional effects (Fisher et al., 2025).

### 3.6 Regulation of DWORF-SERCA interaction

Regulation of the DWORF-SERCA interaction appears to be closely integrated with the classical β-adrenergic pathway through effects on PLN. Phosphorylation of PLN at Ser16 and Thr17 during adrenergic stimulation weakens PLN-SERCA binding, relieving inhibition and facilitating DWORF access to the regulatory binding site (Bovo et al., 2024). In systems where PLN and DWORF are present at roughly equimolar ratios, PLN phosphorylation further shifts the balance toward activation, allowing DWORF to enhance SERCA activity and increase SR Ca^2+^ load. Within DWORF itself, specific N-terminal residues such as Lys4 and His11 modulate binding and activation, and mutations at these positions alter both FRET interactions and functional readouts (Reddy et al., 2022). These findings suggest that the N-terminal amphipathic region helps tune the orientation and affinity of DWORF for SERCA.

Whether DWORF itself is subject to regulation through phosphorylation or other post-translational modifications remains an open question. Similarly, how DWORF oligomerization and interaction networks change during stress or disease is not fully understood. Elucidating these regulatory mechanisms will be important for developing therapeutic strategies that leverage DWORF‐mediated activation of SERCA while preserving physiological control of the SERCA-PLN regulatory axis.

## 4 Functional consequences in vitro and in vivo

### 4.1 Cellular Ca^2+^ handling

Across cellular systems, DWORF consistently enhances SERCA-mediated Ca^2+^ uptake and SR Ca^2+^ load. In cardiomyocytes isolated from DWORF transgenic mice, peak systolic Ca^2+^ transient amplitude and SR Ca^2+^ content are increased, while the decay of the Ca^2+^ transient is accelerated, reflected by a shorter relaxation time constant (tau), without appreciable changes in NCX-mediated Ca^2+^ extrusion (Makarewich et al., 2018; Nelson et al., 2016). These findings indicate that DWORF accelerates SR Ca^2+^ reuptake rather than simply shifting Ca^2+^ removal toward alternative pathways. In contrast, DWORF-knockout muscle fibers exhibit slowed relaxation following tetanic stimulation, consistent with impaired SERCA-dependent Ca^2+^ clearance under conditions of high-demand (Nelson et al., 2016). Heterologous expression systems expressing SERCA with or without PLN further support this interpretation. Introduction of DWORF increases ER Ca^2+^ content and SERCA-dependent Ca^2+^ uptake beyond that observed with SERCA alone or with SERCA plus PLN, indicating that DWORF can both relieve inhibitory tone and directly enhance pump function (Fisher et al., 2021; Li et al., 2021; Makarewich et al., 2018).

### 4.2 *In vivo* cardiac performance

At the whole-organ level, DWORF overexpression produces a hypercontractile yet hemodynamically well-tolerated phenotype (Nelson et al., 2016). DWORF transgenic mice display increased cardiomyocyte fractional shortening, enhanced SERCA2a activity, faster SR Ca^2+^ reuptake and enhanced relaxation kinetics without evidence of pathological hypertrophy or spontaneous heart failure under resting conditions. Interestingly, these animals display a blunted inotropic response to acute β-adrenergic stimulation, such as administration of isoproterenol. This observation suggests that part of the β-adrenergic reserve is effectively converted into a constitutive enhancement of Ca^2+^ cycling via DWORF-mediated SERCA activation (Nelson et al., 2016).

DWORF-knockout mice are similarly viable and do not develop overt heart failure during aging. However, subtle defects in relaxation kinetics are evident in skeletal muscle and may become more apparent in the heart under conditions of physiological or pathological stress. Together, these observations support a model in which DWORF is not strictly required for basal cardiac function but plays an important role in optimizing contractile performance and relaxation when workload increases.

### 4.3 Mitochondrial function and energetics

The functional effects of DWORF extend beyond cytosolic Ca^2+^ handling to influence mitochondrial metabolism. Hearts from DWORF transgenic mice display increased basal, maximal, and spare respiratory capacity, indicating an enhanced ability to generate ATP across a range of workloads without detectable disruption of respiratory chain complex assembly (Brito-Estrada et al., 2025). Both chronic and acute DWORF overexpression enhance mitochondrial Ca^2+^ uptake. In chronic models this is accompanied by increased expression of the mitochondrial Ca^2+^ uniporter (MCU), whereas acute overexpression enhances respiration even in the absence of major transcriptional changes (Brito-Estrada et al., 2025).

These mitochondrial effects are associated with reduced inhibitory phosphorylation of pyruvate dehydrogenase, linking improved SR Ca^2+^ cycling to enhanced oxidative metabolism and more efficient substrate utilization (Brito-Estrada et al., 2025; Glancy and Balaban, 2012). Importantly, a modest but statistically significant increase in ROS-associated markers was reported in DWORF transgenic mice, consistent with the expected consequence of enhanced mitochondrial Ca^2+^ uptake and increased respiratory flux. These changes did not appear to diminish the overall cardioprotective effects of DWORF in this model. However, increased ROS generation could potentially contribute to pathological remodeling in disease settings characterized by pre-existing oxidative stress, such as DMD or ischemia-reperfusion injury, and therefore warrants further systematic evaluation across disease contexts and expression levels.

### 4.4 Effects on pathological remodeling

In several models of cardiac disease, increased DWORF expression mitigates pathological remodeling of the myocardium. Across models of dilated cardiomyopathy, Duchenne muscular dystrophy, pressure overload-induced heart failure, and ischemia-reperfusion injury, DWORF overexpression reduces myocardial fibrosis and attenuates activation of the fetal gene program, including markers such as *Nppa* and *Myh7* (Brito-Estrada et al., 2025; Makarewich et al., 2020; Makarewich et al., 2018; Morales et al., 2023; Stege et al., 2023).

These histological and transcriptional changes parallel improvements in global cardiac function, indicating that DWORF can limit both structural remodeling and molecular reprogramming associated with chronic cardiac stress. However, the rescue is not uniformly complete. In some models, particularly DMD and severe pressure overload, diastolic indices such as tau remain only partially improved, even when systolic function and mitochondrial performance are substantially enhanced (Brito-Estrada et al., 2025; Morales et al., 2023). This pattern suggests that DWORF’s strongest effects involve enhancement of systolic performance and energetic capacity, whereas some aspects of diastolic dysfunction may require additional regulatory mechanisms or therapeutic interventions.

### 4.5 Comparative perspectives: DWORF vs. other SERCA-targeted strategies

It is informative to consider DWORF‐mediated modulation of SERCA alongside other strategies that target the SERCA-PLN regulatory axis (Njegic et al., 2020; Periasamy et al., 2008). Phosphorylation of PLN enhances SERCA activity transiently in response to β-adrenergic stimulation, whereas PLN knockdown or knockout removes inhibitory tone entirely. SERCA2a overexpression increases the abundance of the pump independently of PLN (Jessup et al., 2011). DWORF overexpression differs from these approaches in several important ways. DWORF relieves PLN-mediated inhibition through direct competition, directly increases SERCA catalytic turnover, and biases pump activity toward high-throughput states during periods of elevated Ca^2+^ cycling, all while preserving endogenous β-adrenergic signaling pathways. Consequently, these strategies are not functionally equivalent and produce distinct effects on parameters such as apparent Ca^2+^ affinity (K_Ca_), maximal pump activity (Vmax), diastolic Ca^2+^ levels, and β-adrenergic responsiveness as summarized in Table 2.

**TABLE 2:** Comparative overview of therapeutic strategies targeting the SERCA2a-PLN regulatory axis in heart failure.

Therapeutic strategy	Mechanism	Major functional effects	Preclinical/Clinical evidence	Translational advantages	Key limitations/Safety	References
DWORF overexpression	Direct SERCA2a activation; competitive displacement of inhibitory regulins (PLN, SLN, ALN); biases SERCA toward high-Ca^2+^ states	↑ SR Ca^2+^ uptake; ↑ systolic Ca^2+^ transients; faster relaxation; improved contractility; enhanced mitochondrial energetics	Protective effects demonstrated in multiple mouse models of heart failure, including MLP-KO, TAC, DMD, MI, and PLN-R14del; substantial benefit is observed across models, although the degree of rescue varies by disease context; no clinical studies to date	Short coding sequence is highly compatible with AAV delivery; endogenous positive regulator of SERCA2a; preserves physiologic PLN regulatory network; improves mitochondrial energetics in addition to Ca^2+^ handling	Optimal dosing unknown; long-term safety undefined; potential risk of SR Ca^2+^ overload and arrhythmogenesis; lack of large-animal therapeutic validation; immune and delivery barriers remain unresolved	Nelson et al. (2016), Makarewich et al. (2018), Makarewich et al. (2020), Cleary et al. (2022), Stege et al. (2023), Morales et al. (2023), Brito-Estrada et al. (2025)
PLN phosphorylation (β-adrenergic signaling)	PKA/CaMKII-mediated phosphorylation weakens PLN-SERCA inhibition during adrenergic stimulation	↑ SERCA Ca^2+^ affinity; faster relaxation; increased contractile reserve during stress	Central physiologic mechanism in acute fight-or-flight signaling; extensively characterized experimentally; no clinical studies to date	Physiologic and reversible; dynamically coupled to physiologic demand; preserves inhibitory “brake” on SERCA2a at baseline	Chronic β-adrenergic activation is maladaptive; associated with arrhythmia and remodeling; difficult to selectively target PLN phosphorylation without systemic effects	Brittsan et al. (2000), Glaves et al. (2011), Kranias and Hajjar (2012), Henry et al. (2025)
PLN knockdown/knockout	Removes inhibitory PLN regulation resulting in constitutive SERCA2a disinhibition	Sustained ↑ SR Ca^2+^ load; enhanced baseline contractility; faster relaxation	PLN-null mice exhibit hypercontractility; human PLN loss-of-function mutations associated with severe DCM and arrhythmogenic disease; no clinical studies to date	Potent enhancement of SERCA activity; conceptually simple target	Loss of physiologic SERCA regulation; potential for arrhythmia and maladaptive remodeling; human genetic data raise substantial safety concerns	Luo et al. (1994), MacLennan and Kranias (2003), Haghighi et al. (2003), Haghighi et al. (2014)
SERCA2a overexpression	Increases SERCA2a protein abundance independent of PLN status	↑ SR Ca^2+^ reuptake capacity; improved systolic and diastolic function	Robust benefit in many animal models of HF; mixed/negative results in clinical trials (CUPID2), likely due to inefficient SERCA2a gene transfer	Direct targeting of SERCA2a; broad mechanistic applicability across HF etiologies	Large cDNA challenges AAV packaging capacity; vector delivery limitations; mixed clinical efficacy; potential energetic and arrhythmogenic concerns at high expression	Periasamy et al. (2008), Gwathmey et al. (2011), Gorski et al. (2015), Greenberg et al. (2016), Zhihao et al. (2020)

Comparison of major therapeutic approaches aimed at enhancing SERCA2a function or relieving PLN-mediated inhibition in heart failure, including DWORF overexpression, PLN phosphorylation, PLN knockdown/knockout, and SERCA2a overexpression. Strategies are compared based on mechanism of action, major functional effects on Ca^2+^ handling, key preclinical and clinical findings, translational advantages, and important limitations or safety considerations. Collectively, these approaches illustrate both the therapeutic potential and translational challenges associated with modulating SR Ca^2+^ cycling in the failing heart.

## 5 DWORF in disease models and preclinical gene therapy

**FIGURE 2:**
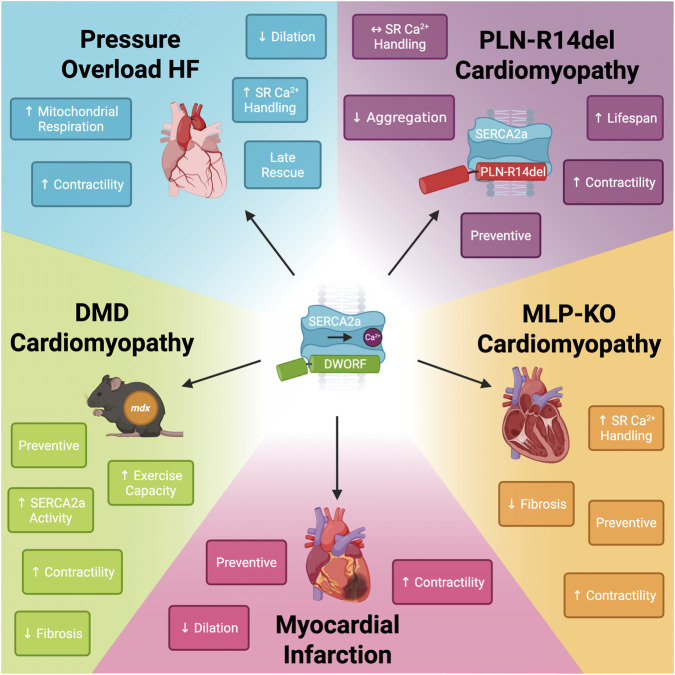
Schematic overview of preclinical disease models in which DWORF has demonstrated therapeutic benefit. Each sector represents a distinct disease context with the delivery strategy, key outcomes, and important caveats indicated in each model. In pressure overload-induced HF, cardiotropic MyoAAV-mediated DWORF delivery attenuated ejection fraction decline, reduced LV dilation, and enhanced mitochondrial respiration in both a preventative and late rescue strategy for transverse aortic constriction, highlighting the benefits of DWORF even at a stage of established disease. In PLN-R14del cardiomyopathy, transgenic DWORF overexpression extended lifespan, reduced pathological PLN protein aggregate burden, and improved echocardiographic and electrocardiographic parameters despite only minimal changes in SR Ca^2+^ handling. In MLP-KO cardiomyopathy, transgenic DWORF overexpression or AAV9-DWORF normalized SR Ca^2+^ handling, reduced fibrosis, and improved contractility in a genetic model of dilated cardiomyopathy. In DMD cardiomyopathy, systemic AAV9-mediated delivery of DWORF in aged *mdx* mice increased SERCA2a activity, improved contractility and exercise capacity, and reduced myocardial fibrosis. In myocardial infarction, systemic AAV9-mediated DWORF delivery preserved ventricular function, attenuated LV dilation, and maintained SR Ca^2+^ uptake following coronary ligation. Tg, transgenic overexpression; MyoAAV, muscle-tropic adeno-associated virus; AAV9, adeno-associated virus serotype 9; SR, sarcoplasmic reticulum; DMD, Duchenne muscular dystrophy; MLP-KO, muscle LIM protein knockout; PLN, phospholamban; HF, heart failure. See Table 3 for additional details relevant to this figure.

The evidence supporting DWORF as a therapeutic target spans several preclinical disease models encompassing genetic dilated cardiomyopathies, muscular dystrophy, and acquired heart failure etiologies with each interrogated using either transgenic overexpression or AAV-mediated gene delivery (Figure 2; Table 3). Across these contexts, DWORF consistently enhances SERCA2a activity, attenuates structural remodeling, and improves contractility, while the mechanistic basis of benefit and degree of rescue vary meaningfully by disease setting. The following sections examine each model in detail, highlighting both the therapeutic promise and the disease-specific limitations that will inform future translational efforts.

**TABLE 3:** Comparative summary of preclinical studies evaluating DWORF overexpression as a therapeutic strategy in cardiac disease (related to Figure 2).

Disease model	Species/System	Delivery strategy (Promoter)	Overexpression level	Treatment timing	Key outcomes	Translational relevance	Limitations/Unanswered questions	References
MLP-KO Cardiomyopathy	Mouse (MLP −/−)	DWORF-Tg (αMHC); AAV9-DWORF (cTnT)	Tg: ∼60-fold; AAV: ∼17-fold	Preventive	↑ SR Ca^2+^ transient amplitude and SR load ↑ EF and FS (Echo) ↓ fibrosis ↑ mitochondrial respiration; attenuation of fetal gene program	Demonstrates efficacy in a genetic dilated cardiomyopathy model with severe Ca^2+^ dysregulation	AAV delivered neonatally; no direct human MLP-null equivalent disease; long-term safety not assessed	Arber et al. (1997), Makarewich et al. (2018), Makarewich et al. (2020)
DMD Cardiomyopathy	Mouse (*mdx*, aged; 18 months)	AAV9-DWORF (CMV)	∼50-fold	Preventive	↑ SERCA2a Vmax ↑ EF and maximal pressure (Hemo) ↑ exercise capacity ↑ ECG amplitude ↔ LV tau	Supports potential application in dystrophic cardiomyopathy and skeletal muscle disease	Ubiquitous promoter may produce off-target expression; incomplete normalization of cardiac function; arrhythmia outcomes not assessed	Hoffman et al. (1987), Morales et al. (2023), Morales et al. (2024), Gibson et al. (2025)
Pressure Overload Heart Failure	Mouse (TAC)	MyoAAV-DWORF (cTnT)	∼25-fold	Preventive and Late Rescue	↑ Contractility (Echo, IonOptix) ↑ SR Ca^2+^ transient amplitude (late rescue) ↓ fibrosis (late rescue); modest ↑ ROS	Most clinically relevant gene therapy study because efficacy was demonstrated in established disease	Modest increase in ROS; arrhythmia susceptibility not examined; effects of chronic DWORF expression remain unknown	Richards et al. (2019), Brito-Estrada et al. (2025)
PLN-R14del Cardiomyopathy	Mouse (PLN-R14del knock-in)	DWORF-Tg (αMHC)	∼100-fold	Preventive	↑ Survival ↑ EF (Echo) ↓ fibrosis ↓ PLN aggregation ↔ SR Ca^2+^ handling; attenuation of fetal gene program	Direct relevance to inherited human PLN cardiomyopathy	Mechanism of benefit remains incompletely defined; SR Ca^2+^ handling largely unchanged despite functional improvement; interaction between DWORF and mutant PLN requires further study	Stege et al. (2023), Stege et al. (2024a), Maas et al. (2025)
Myocardial Infarction	Mouse (LAD ligation)	AAV9-DWORF (cTnT)	∼17-fold	Preventive	↑ FS (Echo) ↓ Dilation (Echo) ↔ Infarct size	Supports utility across acquired forms of heart failure	No mechanistic studies performed; no reduction in infarct size or myocyte death; therapeutic efficacy after established infarction remains unknown	Reichert et al. (2017), Makarewich et al. (2020)

Studies are compared by disease model, experimental design, timing of intervention, degree of DWORF overexpression, principal efficacy outcomes, translational relevance, and major limitations. Collectively, these studies demonstrate that DWORF consistently improves cardiac function and remodeling across diverse disease settings, although the magnitude and mechanistic basis of rescue vary considerably between models. Outcome direction is indicated as ↑ (increase), ↓ (decrease), or ↔ (no significant change). MLP, muscle LIM protein; DMD, Duchenne muscular dystrophy; TAC, transverse aortic constriction; LAD, left anterior descending coronary artery; Tg, transgenic; AAV, adeno-associated virus; αMHC, alpha-myosin heavy chain; cTnT, cardiac troponin T; CMV, cytomegalovirus; SR, sarcoplasmic reticulum; EF, ejection fraction; FS, fractional shortening; Echo, echocardiography; Vmax, maximal pump turnover rate; Hemo, hemodynamics; ECG, electrocardiography; LV, left ventricle; Tau, relaxation time constant; ROS, reactive oxygen species.

### 5.1 Dilated cardiomyopathy: the MLP-knockout model

Muscle LIM protein knockout (MLP KO) mice (null for the *Csrp3* gene) are a well-established model of dilated cardiomyopathy characterized by sarcomere disorganization, Ca^2+^ mishandling, fibrosis, and progressive heart failure (Arber et al., 1997). Earlier studies showed that crossing MLP KO mice with PLN KO animals markedly improved cardiac function, suggesting that augmenting SERCA2a activity through relief of PLN-mediated inhibition is sufficient to blunt many features of disease in this model (Minamisawa et al., 1999). However, subsequent human genetic studies revealed that complete loss of PLN is deleterious in patients, highlighting important species-specific differences and limiting the translational appeal of PLN ablation as a therapeutic strategy (Haghighi et al., 2003).

Nearly 3 decades later, crossing DWORF transgenic mice into the MLP KO background produced a similarly striking rescue of the cardiomyopathic phenotype, with normalization of Ca^2+^ handling and substantial attenuation of structural remodeling (Makarewich et al., 2018). Unlike PLN knockout, DWORF enhances SERCA2a function through a modulatory activation rather than elimination of an essential regulatory brake, raising the possibility of a more tunable and potentially safer approach in humans. A follow up study using cardiac troponin T (cTnT) driven AAV9-DWORF delivered to postnatal day 5 pups at 5 × 10^13^ vector genomes per kilogram demonstrated similar prevention effects with a ∼17-fold increase in DWORF compared to the ∼60-fold increase seen in DWORF transgenic mice (Makarewich et al., 2020). Together, the PLN KO and DWORF overexpression studies in MLP-deficient hearts underscore the therapeutic potential of boosting SERCA2a activity in structurally driven DCM, while positioning DWORF as a more translationally promising means of achieving benefits similar to those originally observed with PLN ablation.

### 5.2 DMD cardiomyopathy

Duchenne muscular dystrophy (DMD) provides another important setting in which to test the therapeutic potential of DWORF, particularly in the context of secondary cardiomyopathy (Hoffman et al., 1987). In dystrophin-deficient *mdx* mice and in large-animal DMD dog models, DWORF expression is reduced in the heart and in select skeletal muscles (Gibson et al., 2025; Morales et al., 2024; Morales et al., 2023). In the canine model, transcript and protein analysis indicate a decline in DWORF levels in skeletal muscle as well as in the left ventricle and right atrium beginning as early as 8–13 months and persisting through symptomatic stages. These changes occur alongside dysregulation of other regulins and SERCA isoforms and may show sex-specific patterns, with females generally exhibiting lower DWORF, SERCA2a, and PLN expression (Morales et al., 2024).

Gene therapy experiments in aged *mdx* mice demonstrate that restoration of DWORF can meaningfully improve the dystrophic heart. Systemic AAV9 delivery of DWORF driven by a cytomegalovirus (CMV) promoter at 6 × 10^12^ vector genomes per mouse produces robust myocardial overexpression, on the order of ∼50-fold (Morales et al., 2023). At this dosing, SERCA2a Vmax is increased without major changes in the expression of other Ca^2+^-handling proteins, and global cardiac function improves (Morales et al., 2023). Exercise capacity, measured by treadmill endurance, is also enhanced, and myocardial fibrosis is reduced. Notably, the relaxation constant tau remains impaired, indicating that although DWORF substantially improves systolic function and prevents structural remodeling, diastolic dysfunction is only partially corrected (Morales et al., 2023). It is important to note that the use of a ubiquitous CMV promoter in this study raises the possibility that DWORF expression in skeletal muscle or other non-cardiac tissues contributed to some aspects of the observed phenotype. Expression in non-cardiac tissues was not systematically evaluated, making it difficult to distinguish direct myocardial benefits from potential contributions of skeletal muscle DWORF expression to the improved exercise capacity. This distinction may be therapeutically relevant, as DWORF expression in the soleus muscle has also been reported to be reduced in *mdx* mice (Morales et al., 2023). Together, these findings support the concept that DWORF gene therapy could complement microdystrophin or other dystrophin-restoring approaches to more fully address the cardiac manifestations of DMD.

### 5.3 Pressure overload-induced heart failure

Transverse aortic constriction (TAC) models pressure overload-induced heart failure with combined systolic and diastolic dysfunction as well as sustained energetic stress, making it an informative context for testing DWORF’s capacity to support the failing ventricle (Richards et al., 2019). In adult mice subjected to TAC, cardiotropic MyoAAV-mediated DWORF overexpression at a dose of 4 × 10^13^ vector genomes per kilogram produces a ∼25-fold increase in DWORF and both attenuates the progressive decline in ejection fraction and limits left ventricular dilation, as reflected by smaller increases in LV internal dimensions (Brito-Estrada et al., 2025; Tabebordbar et al., 2021). At the cellular level, cardiomyocytes isolated from DWORF-treated TAC hearts show improved contractility and trends toward faster Ca^2+^ reuptake compared with control vector-treated counterparts (Brito-Estrada et al., 2025).

Mitochondrial analyses further show that the TAC-induced decline in respiratory capacity is attenuated by DWORF, with preserved or enhanced basal and maximal oxygen consumption and improved pyruvate dehydrogenase activation (Brito-Estrada et al., 2025). These observations are further supported by experiments in human induced pluripotent stem cell -derived cardiomyocytes (hiPSC-CMs), in which increasing DWORF dosage produces dose-dependent improvements in mitochondrial respiration, suggesting that the coupling between DWORF-mediated SERCA activation and mitochondrial metabolism is conserved in human cells (Brito-Estrada et al., 2025).

A particularly translationally relevant aspect of this work is the demonstration of “late rescue.” When MyoAAV-DWORF is administered 6 weeks after TAC, at a stage when substantial structural and molecular remodeling has already occurred, DWORF still improves ejection fraction and mitochondrial function, with little effect on lung or heart weight (Brito-Estrada et al., 2025). Whereas many cardiac gene therapy studies focus on prevention or early intervention, DWORF joins a relatively small group of targets, including Junctophilin-2 (JPH2) and cardiac Bridging Integrator 1 (cBIN), for which AAV-mediated expression has demonstrated rescue of established cardiac dysfunction in stringent animal models (Khan et al., 2025; Reynolds et al., 2016). The combination of systemic delivery, cardiomyocyte-specific overexpression, improved Ca^2+^ cycling, and restoration of mitochondrial energetics highlights DWORF as an especially promising candidate for translation among SERCA-modulating strategies.

### 5.4 PLN-R14del cardiomyopathy

Phospholamban R14del (PLN-R14del) cardiomyopathy is a highly penetrant genetic form of dilated and arrhythmogenic cardiomyopathy characterized by early ventricular arrhythmias, progressive systolic dysfunction, and elevated risk of sudden cardiac death (Stege et al., 2024a). Mouse models carrying the murine PLN-R14del mutation recapitulate many aspects of the human phenotype. Homozygous PLN-R14del mice develop rapid-onset heart failure with marked PLN aggregation in perinuclear SR/ER clusters, profound remodeling, and early mortality (Eijgenraam et al., 2020). These aggregates also sequester SERCA2a and other SR proteins, suggesting that toxicity may reflect not only altered SERCA binding kinetics but also protein mislocalization and proteotoxic stress (Eijgenraam et al., 2021). Heterozygous models develop a milder but still progressive cardiomyopathy, with variable degrees of contractile dysfunction, fibrosis, and arrhythmia over time (Maniezzi et al., 2024).

DWORF has emerged as a modifier of PLN-R14del cardiomyopathy in preclinical studies. Crossing transgenic DWORF overexpressing mice with homozygous PLN-R14del knock-in mice extends lifespan, delays the onset of overt heart failure, and improves both echocardiographic and electrocardiographic parameters compared to PLN-R14del mice alone (without DWORF overexpression) (Stege et al., 2023). In these compound mice, DWORF markedly reduces the burden of PLN aggregates and associated SR/ER clusters, suggesting that some of its benefit may arise from limiting aggregate formation rather than solely from classical relief of SERCA inhibition (Stege et al., 2023). Notably, in this context, DWORF produces only modest early changes in cellular Ca^2+^ handling, consistent with a predominantly proteostatic or trafficking related effect in the context of severe PLN pathology (Stege et al., 2023).

A related study reported a similar reduction in aggregate burden with overexpression of PLN-L31A/I40A, a truncated PLN mutant that retains high affinity for SERCA2a but lacks inhibitory function (Chen et al., 2026). This finding raises the possibility that competition for SERCA2a binding reduces formation of the distinctive PLN-R14del aggregates. Together, these studies suggest that DWORF’s high affinity for SERCA2a and its ability to compete with PLN-R14del may contribute to reduced pathologic aggregation, although this mechanistic link will require further direct validation.

Complementary studies in PLN-R14del heterozygous mice lacking endogenous DWORF indicate that loss of DWORF does not dramatically worsen cardiac function, fibrosis, or aggregate formation under baseline or early disease conditions (Stege et al., 2024b). These findings suggest that endogenous DWORF is not strictly required for survival in the setting of PLN-R14del heterozygosity, but that increased DWORF expression can provide meaningful, albeit incomplete, protection in more severe disease states (Maas et al., 2025). Overall, the PLN-R14del models illustrate both the promise and the limits of DWORF-based therapies in a setting characterized by severe SERCA dysregulation and protein aggregation, underscoring the importance of disease context when considering DWORF as a therapeutic target.

### 5.5 Ischemia-reperfusion and oxidative stress

Ischemia-reperfusion (I/R) injury is a setting in which oxidative stress, Ca^2+^ overload, and impaired SERCA function converge (Altamirano et al., 2015). In murine models of myocardial I/R, expression of both SERCA2a and DWORF is reduced in the infarct border zone, coinciding with impaired Ca^2+^ handling and contractile dysfunction (Asensio-Lopez et al., 2025; Makarewich et al., 2020). Treatment with the ROS-scavenging compound AEOL activates NRF2 signaling and restores SERCA2a activity, as well as the physical interaction between DWORF and SERCA, suggesting that this axis may represent one downstream effector of antioxidant-mediated cardioprotection (Asensio-Lopez et al., 2025).

Related work in an air pollution associated cardiomyopathy model found reduced NRF2 and DWORF expression in the hearts of Wistar rats exposed to particulate pollutants, with reversal of these changes following treatment with curcumin, a potent antioxidant derived from turmeric (El Tabaa et al., 2022). The functional connection between NRF2, DWORF, and antioxidant treatment was further supported by experiments in NRF2-knockout mice and human iPSC-derived cardiomyocytes with NRF2 suppression. In these settings, AEOL treatment failed to rescue SERCA activity or DWORF-SERCA interactions unless exogenous DWORF was supplied (Asensio-Lopez et al., 2025).

Complementary studies using AAV-mediated DWORF gene transfer in a permanent coronary ligation model further support a protective role for DWORF in ischemic heart failure. In this study, mice received systemic AAV-DWORF at 5 × 10^13^ vector genomes per kilogram early in life with a cardiac restricted cTnT promoter and were subjected to myocardial infarction in adulthood (Makarewich et al., 2020). Endogenous DWORF expression declined after MI, whereas DWORF overexpression of ∼17-fold was achieved and significantly improved ventricular function, limited LV dilation, and preserved SR Ca^2+^ uptake despite similar infarct size compared with control-treated animals (Makarewich et al., 2020). Collectively, these findings position DWORF as an important node linking redox signaling, SERCA function, and functional recovery after ischemic injury, and they support DWORF gene therapy as a promising strategy to mitigate both acute I/R damage and chronic post-MI remodeling.

### 5.6 Sex and hormonal regulation of DWORF

Sex hormones and menopausal status may modulate the SERCA-PLN-DWORF axis, although current data are limited and illustrate several broader challenges in microprotein biology (Regitz-Zagrosek and Kararigas, 2017). In a 4-VinylCyclohexene Diepoxide (VCD)-induced perimenopause model, investigators observed a progressive decline in SERCA2a activity accompanied by changes in regulin expression (Barry et al., 2024). During the perimenopausal window, SLN, MLN, and DWORF levels increased, whereas in a menopause-like state, DWORF expression fell below that of age-matched controls, coinciding with impaired Ca^2+^ uptake and altered NCX expression.

Similarly, work in large-animal DMD models suggests sex-dependent differences in the expression of DWORF, SERCA2a, and PLN in both heart and skeletal muscle, raising the possibility that hormonal milieu and sex chromosomes may influence regulin expression over time (Morales et al., 2024). Together, these findings suggest that DWORF may participate in sex-specific responses to chronic stress and cardiometabolic perturbation, but they also underscore the need for additional work before firm conclusions can be drawn.

Collectively, these studies demonstrate that the magnitude and nature of DWORF-mediated rescue vary substantially across disease contexts. The most robust improvements have been observed in pressure-overload and MLP-deficient models, where DWORF improves contractility, mitochondrial function, and structural remodeling. In contrast, rescue in DMD and PLN-R14del cardiomyopathy appears more partial and disease-context-dependent, with persistent abnormalities in relaxation kinetics, calcium handling, or aggregate-associated pathology despite meaningful functional benefit. These differences likely reflect disease-specific mechanisms, timing of intervention, expression level, and the relative contribution of SERCA dysregulation to the underlying pathology.

## 6 Human and translational considerations

### 6.1 Bioinformatic analysis of DWORF in human heart failure datasets

Large-scale transcriptomic resources available through public repositories such as GEO and ArrayExpress provide an opportunity to analyze DWORF expression within the broader context of human heart failure. However, several technical considerations complicate quantitative interpretation. DWORF is encoded by an unusually short transcript, which can reduce cDNA yield during library preparation and decrease detection sensitivity relative to longer transcripts, particularly in datasets with modest sequencing depth. Despite these limitations, reanalysis of bulk left ventricular RNA sequencing comparing non-failing hearts with ischemic cardiomyopathy, non-ischemic dilated cardiomyopathy, and hypertrophic cardiomyopathy may help determine whether reduced DWORF expression represents a consistent feature of failing human myocardium. Such analyses could also provide insight into whether DWORF dysregulation is shared across multiple heart failure etiologies or reflects disease-specific remodeling processes.

Additionally, further validation of DWORF downregulation should be conducted using proteomic datasets from human heart failure samples. Traditionally, proteomic approaches have had limited sensitivity for detecting microproteins because standard workflows are inherently biased toward larger proteins following enzymatic digestion and peptide fragmentation. However, recent methodological advances, including microprotein enrichment strategies prior to mass spectrometry, have substantially improved the detection and quantification of these small proteins (Brito-Estrada et al., 2022; Cassidy et al., 2023; Hassel et al., 2023; Valdivia-Francia and Sendoel, 2024). Furthermore, because DWORF was only recently discovered and characterized, it was not included as a target in many earlier proteomic analyses. As a result, existing datasets may need to be reanalyzed, or new studies performed using optimized sample preparation workflows that preserve and enrich microproteins to accurately assess DWORF abundance in human heart failure specimens (Makarewich and Olson, 2017; Valdivia-Francia and Sendoel, 2024).

Similar limitations apply to the assessment of microproteins such as DWORF in single-cell and single-nuclear RNA sequencing datasets of human myocardium. Because these approaches rely on stochastic transcript capture, low-abundance and short transcripts are particularly susceptible to dropout events, resulting in frequent false-negative observations even in cells where the gene is genuinely expressed. Analyses of published human cardiac single-cell atlases have identified DWORF expression predominantly within cardiomyocytes; however, these findings should be interpreted cautiously given the technical challenges associated with detecting low-abundance microprotein transcripts (Chaffin et al., 2022; Litvinukova et al., 2020). Continued improvements in transcript capture efficiency, amplification methods optimized for short transcripts, and complementary approaches such as spatial transcriptomics may ultimately provide a more accurate and comprehensive understanding of DWORF expression patterns at single-cell resolution.

### 6.2 Species differences and human translational relevance

Translating DWORF-based therapies from mouse to human requires careful consideration of species-specific difference in Ca^2+^ handling (Vangheluwe et al., 2005). Mice have exceptionally high heart rates (∼500–600 bpm), short action potentials, and a particularly strong reliance on SERCA-mediated SR Ca^2+^ reuptake to drive cardiac relaxation, whereas humans and large animals operate at lower heart rates with longer action potentials and a relatively greater contribution of NCX to Ca^2+^ removal (Edwards and Louch, 2017; Milani-Nejad and Janssen, 2014). Despite these differences, SERCA2a remains central to ventricular relaxation and contractility across species, and PLN is an important regulator in each setting (Sitsel et al., 2019).

Since DWORF enhances the activity of endogenous SERCA rather than simply increasing SERCA abundance, its mechanism may translate favorably across species by modulating an existing regulatory system rather than introducing supraphysiologic amounts of a large membrane ATPase (Gwathmey et al., 2011). Human iPSC-derived cardiomyocytes (hiPSC-CMs) provide an intermediate experimental platform supporting this idea (van Mil et al., 2018). In hiPSC-CMs with increasing overexpression of DWORF, dose-dependent improvements in mitochondrial respiratory capacity were observed, suggesting that the coupling between DWORF-mediated SERCA activation and enhanced oxidative metabolism observed in rodents is conserved in human cardiomyocytes (Brito-Estrada et al., 2025). An important caveat is that hiPSC-CMs vary substantially in electrophysiologic and structural maturity, and standard differentiation protocols yield cells that more closely resemble fetal or neonatal cardiomyocytes than adult ventricular myocardium (Seibertz et al., 2023). Since DWORF expression increases postnatally during cardiac maturation, its functional context in immature hiPSC-CMs may not fully reflect that in adult myocardium (Nelson et al., 2016; Reddy et al., 2022). Future studies using more mature hiPSC-CM preparations, including metabolic maturation, extended culture, or three-dimensional engineered heart tissue formats, will be important for more fully recapitulating the adult cellular environment in which DWORF normally operates (Feyen et al., 2020; Ronaldson-Bouchard et al., 2018).

### 6.3 Gene therapy design considerations

DWORF’s compact coding sequence offers a practical advantage for gene therapy. The DWORF coding sequence is only ∼105 bp, which fits easily within the packaging limits of AAV vectors and leaves substantial room for regulatory elements or additional transgenes (Zincarelli et al., 2008; Nelson et al., 2016). This small size facilitates efficient AAV packaging, supports high functional titers, and opens the possibility of multiplexed constructs, for example, co-delivery of DWORF with microdystrophin in DMD, or with other modifiers of Ca^2+^ handling and structural integrity (Duan, 2018).

Vector cassette design will be critical for balancing efficacy and safety. Early proof-of-concept studies used ubiquitous promoters such as CMV or CAG (CMV, chicken beta-Actin, rabbit beta-Globin synthetic promoter), or hybrid CMV/chicken β-actin promoters to drive robust DWORF expression in mouse hearts, achieving marked overexpression and clear functional benefit (Morales et al., 2023). For translational applications, however, cardiac-specific promoters will continue to be pursued (for example, cTnT, α-MHC, or engineered cardiomyocyte-restricted elements) to limit off-target expression in non-cardiac tissues (Brito-Estrada et al., 2025; Makarewich et al., 2020).

Tuning promoter strength and vector dose will be essential to avoid excessive SR Ca^2+^ loading, arrhythmia, or unanticipated toxicity. Current preclinical programs are therefore exploring multiple different AAV cassettes and dose levels to define a therapeutic window that maximizes benefit while maintaining long-term safety.

### 6.4 DWORF in the broader Ca^2+^-handling therapeutic landscape

DWORF-based interventions should be viewed within the broader context of therapies that affect cardiac Ca^2+^-handling. Standard HF therapies, including β-blockers, RAAS (Renin-Angiotensin-Aldosterone System) inhibitors, SGLT2 (Sodium-Glucose Cotransporter 2) inhibitors, and myosin modulators, improve outcomes in part by reducing neurohormonal drive, optimizing loading conditions, or modulating myofilament function, but they do not directly correct SR Ca^2+^ cycling (Heidenreich et al., 2022; McMurray et al., 2019). By enhancing SERCA activity and linking improved Ca^2+^ handling to mitochondrial energetics, DWORF could potentially complement these therapies by improving intrinsic contractile efficiency in parallel with established neurohormonal and hemodynamic treatments.

There is also potential for combinatorial gene-based strategies. For example, combining DWORF with SERCA2a overexpression might permit lower levels of SERCA2a transgene expression while preserving or enhancing functional benefit, potentially reducing concerns related to viral toxicity or off-target effects (Gwathmey et al., 2011). Similarly, as previously mentioned, co-delivery of DWORF with microdystrophin in DMD could address both the primary structural defect and the downstream abnormalities in Ca^2+^ handling and energetics (Duan, 2018).

### 6.5 Safety considerations

Any therapy that enhances SR Ca^2+^ uptake must be evaluated carefully for potential safety liabilities. For DWORF, key concerns include the possibility of arrhythmia if SR Ca^2+^ loading becomes excessive, incomplete correction of diastolic dysfunction in some models, and potential off target effects depending on vector design and route of delivery. Peptide infusion studies in isolated rat hearts have also reported vasoconstrictive effects mediated by L-type Ca^2+^ channels and Rho kinase, arguing against systemic administration of free peptide (Mbikou et al., 2020). Gene-based strategies may avoid this issue by restricting DWORF expression to cardiomyocytes, but careful optimization of vector dose and long-term monitoring will still be essential.

Beyond the risk of arrhythmia, excessive SERCA activation can increase SR Ca^2+^ content to levels that predispose to spontaneous SR Ca^2+^ release events, which generate a transient inward current via NCX, producing delayed afterdepolarizations (DADs) and triggered activity that can initiate ventricular arrhythmia (Luo and Anderson, 2013). Dedicated telemetry-based arrhythmia surveillance and programmed stimulation protocols will therefore be important components of future large-animal safety studies. Additionally, chronic elevation of cytosolic Ca^2+^ activates downstream signaling pathways that are established mediators of pathological remodeling in other settings. Calcineurin, a Ca^2+^/calmodulin-dependent phosphatase, responds to sustained cytosolic Ca^2+^ elevation by dephosphorylating and activating NFAT transcription factors, a pathway that drives pathological hypertrophy and fetal gene program in multiple cardiac disease models (Wilkins et al., 2004; Molkentin et al., 1998). CaMKII is similarly activated by elevated Ca^2+^ cycling and mediates RyR2 hyperphosphorylation, increased diastolic SR Ca^2+^ leak, and activation of pro-hypertrophic gene programs (Luo and Anderson, 2013). The available evidence indicates that DWORF transgenic mice do not develop spontaneous hypertrophy or heart failure and that DWORF expression suppresses rather than induces the fetal gene program in disease models (Makarewich et al., 2018; Stege et al., 2023). However, whether the level of SERCA activation achieved by therapeutic DWORF expression is sufficient to chronically engage calcineurin-NFAT or CaMKII signaling under conditions of combined hemodynamic stress, as in aging or hypertrophic cardiomyopathy where these pathways may already be active, has not been systematically tested and represents an important safety consideration to address in future studies.

Compared with SERCA2a overexpression and PLN knockdown or inhibition, DWORF-based approaches may offer a different balance of specificity and physiological integration (Jessup et al., 2011). SERCA2a overexpression increases overall pump abundance and poses packaging challenges for AAV based delivery, with mixed clinical results to date (Greenberg et al., 2016; Jessup et al., 2011; Yla-Herttuala, 2015). PLN knockdown or ablation removes an important inhibitory brake and may carry safety concerns particularly in light of human genetic data (Gwathmey et al., 2011; Haghighi et al., 2003). By contrast, DWORF modulates an endogenous interaction surface, enhances SERCA turnover particularly under high Ca^2+^ conditions, and remains coupled to normal β-adrenergic control through PLN phosphorylation. These features may permit a more nuanced and context-dependent enhancement of SERCA activity, although DWORF efficacy will likely depend on the integrity of the broader SERCA-PLN regulatory network in a given disease state.

## 7 Discussion

The body of preclinical evidence reviewed here positions DWORF as a mechanistically distinct and therapeutically promising microprotein for heart failure. Several themes emerge from synthesis of this literature that deserve explicit consideration: the resolution of ongoing mechanistic controversies, the technical challenges inherent to microprotein biology, the clinical significance of known DWORF mutations, and the practical requirements for clinical translation.

### 7.1 Mechanistic clarifications

Despite rapid progress, several fundamental aspects of DWORF biology remain incompletely understood and represent areas of active debate. The most significant controversy concerns the relative contribution of direct SERCA activation versus PLN displacement to the net functional effect of DWORF. Early studies emphasized PLN displacement as the primary mechanism, while subsequent reconstitution studies clearly demonstrated direct enhancement of SERCA Vmax in the absence of PLN (Fisher et al., 2021; Reddy et al., 2022). The current consensus supports a dual mechanism involving both direct activation and PLN competition, with their relative contributions likely varying with the stoichiometry of DWORF, PLN, and SERCA. These effects are also likely influenced by disease state, endogenous PLN abundance and phosphorylation status, DWORF expression level, and the degree of SERCA dysfunction present within a given pathological setting. In addition to PLN and SERCA, it is possible that interactions with other unidentified binding partners could further modulate the balance between these mechanisms, as the broader DWORF interactome has not been fully characterized (Haghighi et al., 2014). Resolving these relationships quantitatively in native SR membranes at physiological expression levels remains an important goal.

A second unresolved question concerns the oligomeric state of DWORF in membranes. Evidence for predominantly monomeric SERCA-binding coexists with data supporting stable dimer and tetramer formation (Fisher et al., 2025; Singh et al., 2019). The functional significance of oligomerization as either a reservoir to buffer monomeric DWORF availability or as a distinct regulatory mechanism is not established. Whether these oligomeric states change during cardiac stress or disease, and how the lipid composition of the SR membrane influences DWORF conformation and activity, are questions that will require quantitative biophysical approaches in native membrane environments. Additionally, it remains entirely unknown whether DWORF is subject to post-translational modifications such as phosphorylation, ubiquitination, or lipidation that could influence its localization, stability, or interaction with SERCA and other partners. Systematic profiling of DWORF protein across animal models and biobanked human hearts spanning ischemic, non-ischemic, hypertrophic, and HFpEF contexts will be important for defining the disease-specific changes in DWORF expression and function.

### 7.2 Challenges in microprotein biology

A major challenge in microprotein biology is technical rather than conceptual. Because microproteins are often small, low-abundance, and frequently membrane-associated, they can be particularly difficult to detect and study using conventional protein-based approaches. As a result, commonly used techniques such as western blotting, immunostaining, immunoprecipitation, and proteomic analyses are especially susceptible to nonspecific signals, antibody cross-reactivity, and protein misidentification (Brito-Estrada et al., 2025). These challenges are not unique to DWORF but represent a broader issue across the microprotein field that can complicate conclusions regarding protein expression, localization, abundance, and regulation.

To improve rigor and reproducibility, expression analyses are often most reliable when initially anchored at the transcript level using qPCR or RNA sequencing, where assay specificity is generally easier to establish. Protein-level conclusions should ideally rely on antibodies that have undergone stringent validation, including assessment in knockout or knockdown tissues, peptide competition assays, and demonstration of a single band at the predicted molecular weight under optimized western blotting conditions, as exemplified by Gibson et al. (2025). In many cases, genetically engineered epitope-tagged alleles or carefully controlled tagged expression systems provide an important complementary approach for defining subcellular localization, identifying interaction partners, and assessing dynamic regulation.

As the field continues to mature, the integration of orthogonal validation strategies will be essential for establishing confidence in microprotein biology. Combining transcriptomic analyses, rigorously validated antibodies, targeted proteomic approaches, and genetically tagged models provides a robust framework for studying microproteins such as DWORF and for accurately defining their physiological and pathological functions.

### 7.3 DWORF variants: mechanistic implications

The identification of naturally occurring human DWORF coding variants at structurally critical positions provides an opportunity to connect the mutagenesis-based mechanistic framework directly to human genetic variants, though careful interpretation is warranted. Inspection of population variant databases including gnomAD reveals rare missense variants at several of the residues most important for DWORF function including positions His11, Pro15, Gly21, and Gly25 (Chen et al., 2024; Fisher et al., 2025). Of these, Fisher et al. (2025) directly characterized two gnomAD-catalogued variants, p. Pro15Leu and p. Gly25Asp, in reconstituted SERCA activity assays (Fisher et al., 2025). Consistent with structural predictions, p. Pro15Leu, which eliminates the kink central to DWORF’s activating geometry, converts DWORF into a PLN-like inhibitor of SERCA, while p. Gly25Asp disrupts the GxxxG interface and similarly produces inhibitory behavior (Fisher et al., 2025; Reddy et al., 2022). Variants at His11 and Gly21 have been identified in gnomAD but await functional characterization.

For loss-of function variants, the functional consequence would phenocopy the downregulated state observed in failing myocardium. This is unlikely to be acutely catastrophic on its own based on the characterization of DWORF-knockout mice but could represent a baseline vulnerability that lowers the threshold for Ca^2+^ mishandling under hemodynamic stress or in the context of other predisposing variants (Makarewich et al., 2018; Nelson et al., 2016; Stege et al., 2024b). Gain-of-inhibitory-function variants such as p. Pro15Leu require more nuanced consideration. Because DWORF expression is suppressed alongside disease progression in the failing heart, an inhibitory variant would not simply persist as a constitutive SERCA inhibitor throughout disease as it too would be subject to the same silencing that attenuates wild-type DWORF. The more relevant pathophysiological window for such a variant is therefore likely the pre-clinical and early compensated phase, when DWORF is still expressed at meaningful levels. Rather than standalone high-penetrance cardiomyopathy alleles, DWORF coding variants at functional residues are more plausibly modifiers of disease progression and potentially lower the threshold for heart failure development in carriers of other predisposing variants (Karakikes et al., 2015; Klasfeld et al., 2025). Systematic functional characterization of all coding variants in reconstituted SERCA assays and patient-derived cardiomyocyte models, combined with genetic enrichment analyses in large cardiomyopathy biobanks, will be necessary to determine their clinical significance and any interaction with established HF risk alleles (Ceholski et al., 2012; Fisher et al., 2025; van Mil et al., 2018).

### 7.4 Translational path

Given the promising preclinical results in existing DWORF studies, an important next step will be to test DWORF gene therapy in large-animal models of heart failure, such as pig or canine models of pressure overload, DMD cardiomyopathy, or ischemic injury. These studies should include long-term follow-up with comprehensive arrhythmia surveillance, off-target tissue assessments, and evaluation of immune responses to both the AAV capsid and DWORF transgene (Milani-Nejad and Janssen, 2014; Mingozzi and High, 2013). In parallel, optimization of vector design, including capsid selection, promoter choice, regulatory element configuration, and dose, will be needed to define a safe and effective therapeutic window (Zincarelli et al., 2008). Combination strategies such as DWORF plus microdystrophin for DMD, or DWORF plus SERCA2a or other Ca^2+^-handling modifiers, should be explored preclinically to determine whether synergistic benefit can be achieved without increasing safety risk.

A clear clinical positioning strategy will be required to prioritize the first indications for DWORF-based therapy. DMD-associated cardiomyopathy is an attractive initial target given the high unmet need, existing gene therapy infrastructure, and robust preclinical evidence supporting DWORF’s benefit in *mdx* models (Gibson et al., 2025; Morales et al., 2024; Morales et al., 2023). PLN-R14del cardiomyopathy, where DWORF may mitigate aggregate formation while competing for SERCA2a binding, represents another genetically defined, high-penetrance indication where early intervention in identified carriers could be compelling (Stege et al., 2024a; Stege et al., 2023). Pressure overload heart failure and cardiometabolic HF, where SERCA dysfunction and energetic impairment are prominent, are also logical candidates (Brito-Estrada et al., 2025; Kim et al., 2025). HFpEF with documented SERCA dysregulation may represent a niche where DWORF’s combined effects on Ca^2+^ handling and mitochondrial function could be particularly valuable, although the mechanistic complexity of HFpEF will require careful patient selection and stratified trial design (Kim et al., 2025; Shooshtarian et al., 2025). Whatever indication is pursued first, DWORF gene therapy will likely need to be evaluated as an add-on to current standard-of-care therapy in patients with persistent dysfunction and as an early disease-modifying intervention in high-risk genotypes.

### 7.5 Concluding perspective

Over the past decade, the discovery of DWORF has reshaped our understanding of how SERCA2a activity can be regulated by small transmembrane microproteins. Unlike inhibitory regulins such as PLN and SLN, DWORF enhances SERCA2a function while remaining integrated within the endogenous regulatory network that governs Ca^2+^ cycling in striated muscle. This dual mechanism of both direct SERCA2a activation and competitive PLN displacement, its coupling to beta-adrenergic signaling through PLN phosphorylation, and its ability to link improved Ca^2+^ handling to mitochondrial energetics collectively distinguish DWORF from other SERCA2a-targeting strategies. The compact coding sequence makes it ideally suited for AAV gene therapy, and the demonstration of functional rescue even after established disease in the pressure overload model places DWORF among a small and prominent group of gene therapy targets for heart failure. Despite these promising preclinical findings, important questions regarding optimal dosing, long-term safety, arrhythmia susceptibility, immune responses, and translatability across heart failure etiologies remain unresolved. As the fields of microprotein biology and cardiac gene therapy continue to advance, DWORF represents a compelling example of how small, previously overlooked peptides can exert important effects on fundamental physiological processes. Resolving the remaining mechanistic questions, validating efficacy and safety in large-animal models, and advancing carefully designed clinical trials will ultimately determine whether harnessing this endogenous SERCA2a activator can translate into a new class of therapies for heart failure and related muscle diseases.
